# Honokiol, magnolol and its monoacetyl derivative show strong anti-fungal effect on *Fusarium* isolates of clinical relevance

**DOI:** 10.1371/journal.pone.0221249

**Published:** 2019-09-04

**Authors:** Safa Oufensou, Barbara Scherm, Giovanna Pani, Virgilio Balmas, Davide Fabbri, Maria Antonietta Dettori, Paola Carta, Ismael Malbrán, Quirico Migheli, Giovanna Delogu

**Affiliations:** 1 Dipartimento di Agraria, Sezione di Patologia Vegetale ed Entomologia and Unità di Ricerca Istituto Nazionale di Biostrutture e Biosistemi, Università degli Studi di Sassari, Viale Italia, Sassari, Italy; 2 Istituto CNR di Chimica Biomolecolare, Traversa La Crucca, Sassari, Italy; 3 Consejo Nacional de Investigaciones Científicas y Técnicas (CONICET)—Centro de Investigaciones de Fitopatología (CIDEFI-CIC-UNLP), Facultad de Ciencias Agrarias y Forestales, Universidad Nacional de La Plata, La Plata–Buenos Aires, Argentina; Georg-August-Universitat Gottingen, GERMANY

## Abstract

The antifungal activity of magnolol and honokiol, two naturally occurring hydroxylated biphenyls, and of their synthetic derivatives was evaluated on a collection of representative isolates of *Fusarium oxysporum*, *F*. *solani* and *F*. *verticillioides* of clinical and ecological concern. The tested compounds were proposed as a ‘natural’ alternative to conventional fungicides, even though a larger range of concentrations (5–400 μg/ml) was applied. The activity of magnolol and honokiol was compared with that of terbinafine (0.1–10 μg/ml), and fluconazole (1–50 μg/ml), two fungicides widely used in treating fungal infections on humans. Magnolol showed similar fungicidal activity compared to fluconazole, whereas honokiol was more effective in inhibiting mycelium growth compared to this fungicide on all tested clinical *Fusarium* spp. isolates. Compared to terbinafine, honokiol showed similar antifungal activity when tested on clinical *F*. *solani* isolates, whereas magnolol was less effective at all selected concentrations (5–400 μg/ml). The different position of the phenol-OH group, as well as its protection, explain different *in vitro* activities between magnolol, honokiol, and their derivatives. Furthermore, magnolol showed mycelium dry weight reduction at a concentration of 0.5 mM when tested on a set of agricultural isolates of *Fusaria*, leading to complete inhibition of some of them. Magnolol and honokiol are proposed as efficient and safe candidates for treating clinically relevant *Fusaria*.

## Introduction

Natural occurring polyphenols are a great source of biologically active compounds beneficial to human and animal health [1]. Magnolol (**1**, 5,5’-diallyl-2,2’-dihydroxybiphenyl) and honokiol (**2**, 5,5’-diallyl-2,4’-dihydroxybiphenyl) are two hydroxylated biphenyl-type neolignans and represent the main components of the bark of *Magnolia officinalis*, *M*. *obovata*, *M*. *grandiflora* and *M*. *dealbata* [2] These compounds have long been important substances in traditional Chinese and Ayurvedic medicine due to their wide biological activities [3]. Magnolol **1** and honokiol **2** are chemically stable and commercially available in large amounts at a reasonable price. In the bark of *M*. *officinalis*, magnolol **1** and honokiol **2** were identified in ratios of approximately 4:1; honokiol **2** ranges from 17 to 19 mg/g of extract, whereas magnolol **1** is present in the roots at a concentration of 87–96 mg/g. Although the main source of magnolol **1** and honokiol **2** are species of *Magnolia* growing in China, Japan and South Korea, both compounds are also found in other species of this genus present in Mexico. In Asia, these compounds are used in modern clinical practice, while in the United States and Europe are considered cosmetic additives [3].

The unique pharmacophore structure of magnolol **1** and honokiol **2**, formed by two hydroxylated aromatic rings bridged by a single C-C bond representative of the hydroxylated biphenyl structure, has a crucial role in their biological activity [4]. This structural feature allows the activation of a large number of interactions with the surface of proteins [5].

The spectrum of inflammatory diseases to which they provide relief is broad, including allergic rhinitis, influenza and diarrhoea, but also myocardial infarction and anxiety episodes [3, 6]. The biological activity of magnolol **1** and honokiol **2** is likely to depend on the hydroxyl group located at the biphenyl moiety and the allyl chain in para (magnolol **1** and honokiol **2**) and in ortho (honokiol **2**) position to the phenol-OH group, which adds further features to these molecules, such as anti-oxidative, anti-proliferative, anti-fungal, anti-tumoral, anti-HIV and neuroprotective activities [3, 7–10]. This wide array of positive properties makes the two molecules potential candidates in the search for new drugs effective at treating various inflammatory diseases, such as osteoarthritis or acne, but also for prophylaxis of biofilm-forming streptococci causing caries [11].

In hospitals, multi-resistant bacterial and poorly treatable fungal infections have become a serious problem in recent years [12, 13]. Invasive filamentous fungal infections have a growing incidence in immune-compromised subjects such as AIDS patients, recipients of kidney transplants or people suffering from haematological diseases [14]. Among them, *Fusarium* spp. are common human pathogenic fungi implicated in invasive mycoses and infections. Fusariosis is, after aspergillosis, the second most common mould infection in humans [15, 16]. In healthy humans, *Fusarium* spp., particularly *F*. *verticillioides*, *F*. *solani* and *F*. *oxysporum*, are frequently reported as the cause of dermatological affections such as onychomycoses and paronychia, but they may also induce keratitis episodes, mainly as a consequence of contaminated lens solutions [17]. *F*. *solani* and *F*. *oxysporum* represent the major opportunistic human pathogenic filamentous fungi, being responsible for approximately two-thirds of the reported fusarioses. In immune-compromised patients, *Fusarium* infections lead to disseminated fusarioses, which are frequently fatal. In addition to an often-late diagnosis of *Fusarium* spp. infection, currently administrated antifungal drugs are poorly effective due to the high degree of resistance shown by representatives of this genus [15].

*Fusarium* spp. are present in several ecosystems including agricultural soil, with relevant impact on cereal crops [18, 19]. Among other species, *F*. *graminearum* and *F*. *culmorum* are the main pathogens causing *Fusarium* head blight (FHB) and foot and root rot (FRR) on wheat and other small grains, where they induce yield losses, poor grain quality and contamination with type-B trichothecene mycotoxins [20]. Fungicides bearing an azole unit are widely used for plant and human protection against *Fusarium* spp., as they are generally inexpensive, have a broad-spectrum of action and long stability. However, in recent years, large-scale deployment of azole fungicides in agriculture has been considered as a potential cause of the increasing resistance phenomena found also in human pathogenic fungi [21]. Despite different plant extracts are effective against human fusarioses [22], the scarce availability of natural sources from which large amounts of them can be obtained at stable concentrations hamper their application.

In our previous studies devoted to the search of effective sustainable fungicides in agriculture, we observed antifungal activity of magnolol **1** against *F*. *graminearum* and *F*. *culmorum*, both *in vitro* [23] and *in silico* [24]. Magnolol **1** manifested the highest anti-fungal activity among a wide range of natural occurring phenols, with fungal growth inhibition at concentrations as low as 0.125 mM. Furthermore, magnolol **1** and honokiol **2** are generally recognised as harmless for humans and animals [25]. Cytotoxicity (IC_50_) against normal human lymphocytes was set at 38.6 μM for magnolol **1** and 16.1 μM for honokiol **2** [26]. Both compounds are metabolized by extrahepatic and hepatic pathways, producing mainly glucuronic derivatives. Moreover, magnolol **1** is not phytotoxic at concentrations up to 1.5 mM [23]. Considering that many antifungal drugs exert toxic effects on human cells, these characteristics make magnolol **1** and honokiol **2** excellent candidates as sustainable and commercially available fungicides against human and animal pathogens. However, the poor aqueous solubility of magnolol **1** and honokiol **2**, common in natural polyphenols, has hampered their broad clinical application. In order to overcome this issue, encapsulation of honokiol has been assessed [27] and synthetic derivatives were prepared [28]. Nevertheless, derivatives and improved formulations of these compounds are often limited by toxicity and drug interaction, hence preventing their use in medical practice. The pro-drug approach [29] represents a versatile strategy to improve the bioactivity of such molecules by transformation of the hydroxyl groups of magnolol **1** and honokiol **2** in an ester group with a mono- and diacetyl functional group or in an acetal group with a glucose unit, respectively. These ester or acetal groups would undergo *in vivo* biotransformation trough chemical or enzymatic cleavage, thus favouring the delivery of the active compound with a higher yield.

**Fig 1:**
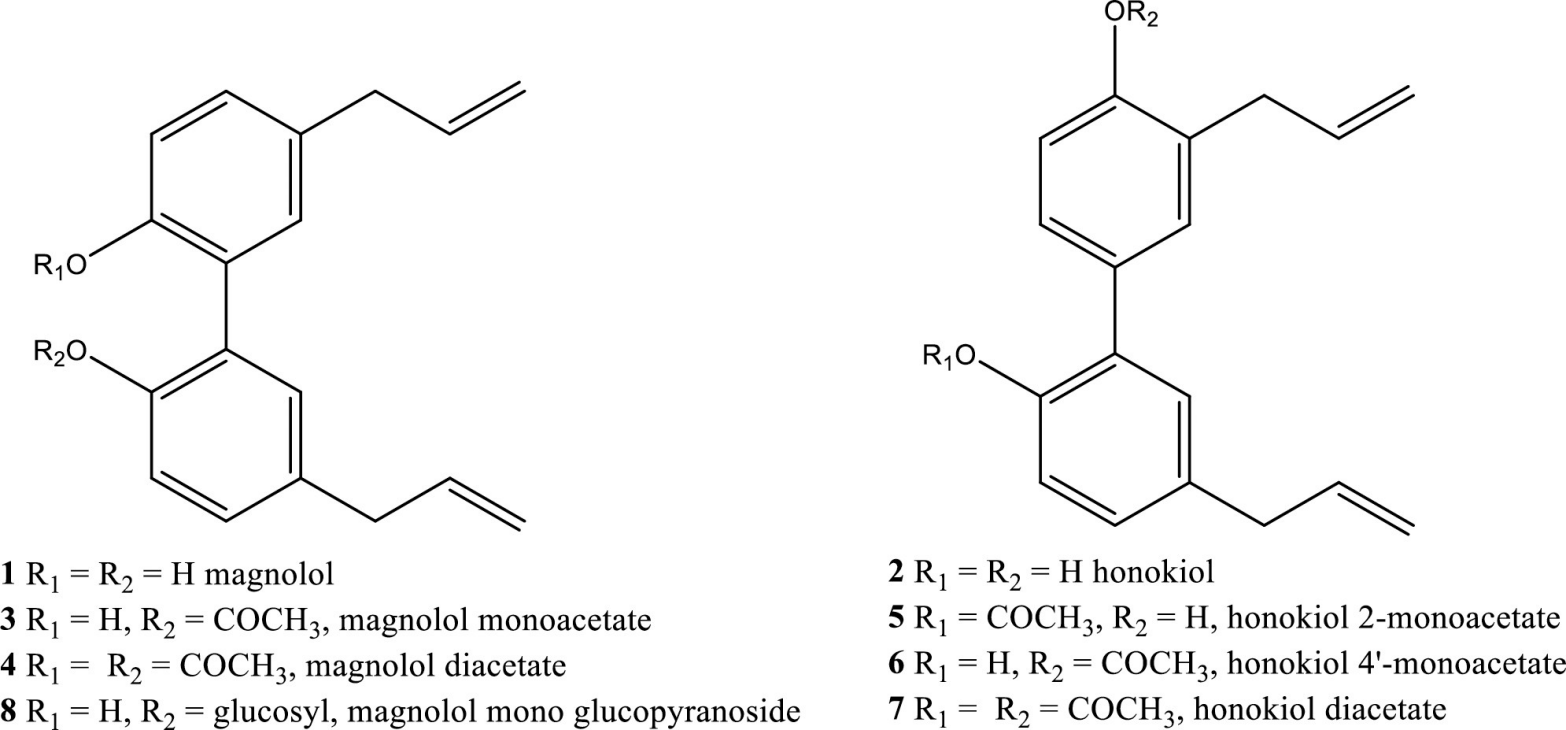
Chemical structures of magnolol 1, honokiol 2 and derivatives 3–8.

Aims of the present work were: 1) to evaluate the inhibitory activity of magnolol **1** and honokiol **2** over a collection of *Fusarium* spp. isolates of clinical and phytopathogenic interest, and 2) to develop derivatives of magnolol **1** and honokiol **2** with improved water solubility and equal inhibitory activity compared to the parental compounds. In a preliminary experiment, the spectrum of activity of magnolol **1** was tested against representative isolates of *Fusarium* species of relevant human concern or of phytopathogenic interest. Subsequently, magnolol **1,** honokiol **2,** and their derivatives (compounds **3**–**8**, Fig 1) were tested for their anti-fungal activity and specificity towards human and nosocomial isolates. The activity of these compounds was compared with that of terbinafine and fluconazole, two common fungicides. To our knowledge, this is the first time that magnolol **1** and honokiol **2** are assayed over a collection of *Fusarium* spp. relevant to both plant and human health.

## Material and methods

### Strains and culture

In a preliminary screening, thirty-two *Fusarium* spp. isolates from the collection of the University of Sassari (PVS-Fu) sampled from soil (10 isolates), diseased plants (9 isolates), and from human specimen (13 isolates) and water tap (1 isolate) in different Italian hospitals were tested against magnolol **1** (Table 1).

**Table 1:** List of the set of 32 *Fusarium* spp. isolates obtained from soil (10 isolates), diseased plants or grain (9 isolates), water tap and human specimen (13 isolates) in different hospitals tested in the preliminary screening.

Species	NRRL n. ^a^	PVS-Fu n.^b^	Diagnosis ^c^	Isolate source	Date	Origin
*F*. *chlamydosporum*	-	4	FHB	durum wheat	1994	Voghera
*F*. *crookwellense*	-	6	FRR	bread wheat	1989	Perugia
*F*. *crookwellense*	-	406	FHB	durum wheat	2008	Sassari
*F*. *guttiforme*	53131	864	Paronychia	finger	2007	Milano
*F*. *langsethiae* ^d^	-	341	FHB	wheat	-	-
*F*. *pseudograminearum*	-	7	FRR	durum wheat	1992	Foggia
*F*. *solani*	46443	93	Dermatomycosis	foot	2004	Milano
*F*. *solani*	46440	97	Onychomycosis	finger	2005	Milano
*F*. *solani*	-	100	Onychomycosis	toe	2006	Milano
*F*. *solani*	52832	95	Onychomycosis	toe	2007	Milano
*F*. *solani*	-	105	Onychomycosis	toe	2010	Milano
*F*. *solani*	44937	107	-	soil	2007	Sassari
*F*. *solani*	46672	108	-	soil	2007	Cagliari
*F*. *solani*	44924	109	-	soil	2007	Cagliari
*F*. *solani*	46492	110	-	soil	2007	Nuoro
*F*. *solani*	46676	111	-	soil	2007	Cagliari
*F*. *subglutinans*	-	10	.	soil	1998	Bari
*F*. *subglutinans*	22034	187	-	maize	-	Iran
*F*. *verticillioides*	-	400	-	maize	2001	Bergamo
*F*. *verticillioides*	53119	112	Sepsis	leukaemia-blood	2007	Ancona
*F*. *verticillioides*	53125	113	Sepsis	kidney transplant-blood	2007	Novara
*F*. *verticillioides*	44894	88	Paronychia	finger	2006	Milano
*F*. *oxysporum*	-	222	-	-	-	-
*F*. *oxysporum*	46489	123	-	soil	2007	Nuoro
*F*. *oxysporum*	46480	124	-	soil	2007	Nuoro
*F*. *oxysporum*	46603	89	Onycomycosis	toe	2004	Milano
*F*. *oxysporum*	46595	126	Onycomycosis	toe	2007	Milano
*F*. *oxysporum*	52682	127	Onycomycosis	toe	2007	Milano
*F*. *oxysporum*	44899	128	Onycomycosis	toe	2006	Milano
*F*. *oxysporum*	-	516	-	water tap	2013	Sassari
*F*. *equiseti*	46660	146	-	soil	2007	Cagliari
*F*. *equiseti*	44916	147	-	soil	2007	Cagliari

^*a*^ NRRL Collection number of Agricultural Research Service (ARS).

^*b*^ PVS-Fu n. Collection number of Dipartimento di Agraria, Sezione Patologia Vegetale ed Entomologia, Sassari, Italy.

^c^ FRR Fusarium Foot Root Rot; FHB Fusarium Head Blight.

^d^ Strains 01–113 kindly provided by Prof. Y. Mattila.

Subsequently, a selection of *F*. *oxysporum*, *F*. *solani* and *F*. *verticillioides* isolates from the PVS-Fu collection, collected from human samples, water tap, and basin sink in different Italian hospitals and identified morphologically and molecularly by analysis of the elongation factor gene sequence (*TEF-1α*) was tested (Table 2).

**Table 2:** List of 15 *Fusarium* spp. isolates of clinical relevance tested in the present study.

n.	Species	NRRL n. ^a^	PVS-Fu n.^b^	Diagnosis	Isolate source	Date	Origin	Hospital^c^
1	*F*. *verticillioides*	-	604	Dead patient	Hospital	2013	Nuoro	NU
2	*F*. *verticillioides*	53129	86	Pneumonia	Broncho alveolar lavage	2007	Varese	V
3	*F*. *verticillioides*	46599	87	Onychomycosis	Toe	2007	Milan	M-1
4	*F*. *verticillioides*	53126	84	Chronic sinusitis	Maxillary sinus pus	2007	Novara	N
5	*F*. *verticillioides*	53122	83	Sepsis in leukemia	Blood	2007	Milan	M-3
**6**	***F*. *verticillioides*** ^**d**^	44894	88	Paronychia	Finger	2006	Milan	M
7	*F*. *solani*	53120	94	Sepsis	Blood	2007	Milan	M-3
8	*F*. *solani*	44903	96	Onychomycosis	Toe	2006	Milan	M-1
9	*F*. *solani*	-	502	-	Sink	2013	Sassari	SS
**10**	***F*. *solani***	46443	93	Dermatomycosis	Foot	2004	Milan	M-1
**11**	***F*. *oxysporum***	46603	89	Onychomycosis	Toe	2004	Milan	M-1
**12**	***F*. *oxysporum***	-	516	-	Water tap	2013	Sassari	SS
13	*F*. *oxysporum*	53121	92	Soft tissue Kaposi’s sarcoma	Pus	2007	Milan	M-2
14	*F*. *oxysporum*	46600	90	Dermatomycosis	Foot	2007	Milan	M-1
15	*F*. *oxysporum*	46606	91	Onychomycosis	Toe	2005	Milan	M-1

^a^ NRRL n. Collection number of Agricultural Research Service (ARS).

^b^ PVS-Fu n. Collection number of Dipartimento di Agraria, Sezione Patologia Vegetale ed Entomologia, Sassari, Italy.

^c^ Locations of eight Italian hospitals sampled listed by city: A, Ancona (Marche region, central Italy); M-1, Sesto San Giovanni Hospital, Milan; M-2, hospital 2, Milan; M-3, hospital 3, Milan; M-4, hospital 4, Milan; V, Varese (Lombardy region, northern Italy); N, Novara (Piedmont region, northern Italy); NU, Nuoro (Sardinia, Italy island); T, Torino (Piedmont region, northern Italy); SS, Sassari (Sardinia, Italy island).

^d^ Isolates in bold were included in the preliminary screening.

### Chemical materials

Magnolol **1** and honokiol **2** were purchased from Chemos GmbH, Germany. Magnolol mono acetate **3**, magnolol diacetate **4**, honokiol 2-mono acetate **5**, honokiol 4’-mono acetate **6** and honokiol diacetate **7** (Fig 1) were prepared as previously described [8]. All compounds were synthesized with the same purity obtained before and judged by ^1^H-NMR spectral determination (in Supporting Information S1 Text). Magnolol mono glucopyranoside **8**, β-anomer, was prepared under chemical and sustainable conditions starting from magnolol-2-*O*-(2’,3’,4’,6’-tetra acetyl)-β-D-glucopyranoside. Structure of **8** was confirmed by comparison with spectroscopic data present in literature of an identical β-anomer achieved under enzymatic conditions (S1 Text in Supporting information).

### Lipophilicity estimation

Lipophilicity was estimated using the logarithm of the partition coefficient for *n*-octanol/water (log P), implemented in the ChemBioDraw Ultra 13.0 software.

### Evaluation of antifungal activity in Vogel’s medium

In a preliminary assay, the antifungal activity of magnolol **1** at 0.5 mM was evaluated according to Pani *et al*. [23] on thirty-two isolates of *Fusarium* spp. (Table 1). Vogel’s medium was amended with 0.5 mM of magnolol **1** and sonicated for one hour at room temperature. Three replicates of 10^4^ conidia in 8 ml of Vogel’s/magnolol **1** medium for each strain were cultured at 25°C in the dark without shaking. After 14 days, mycelia were recovered, filtered and dried at 85°C for 48 h. Inhibition was expressed as dry weight percentage of the untreated test.

## Evaluation of antifungal activity in potato dextrose agar (PDA)

In a second test, the antifungal activity of magnolol **1,** honokiol **2** and their derivatives **3**–**8** (Fig 1) was assessed by comparison with terbinafine and fluconazole, two common fungicides of clinical use, on a selection of *F*. *oxysporum*, *F*. *solani* and *F*. *verticillioides* isolates collected from different Italian hospitals (Table 2). Concentration of compounds **1**–**4** and **8** ranged from 5 to 400 μg/ml, while concentrations between 5–100 μg/ml were used for compounds **5**–**7**. The concentrations of terbinafine and fluconazole ranged from 0.1–10 μg/ml and 1–50 μg/ml, respectively. Antifungal activity was measured after three days of growth on potato dextrose agar (PDA) medium at 25°C in the dark in the presence of the different concentrations (μg/ml) of each compound and expressed as the colony diameter in percentage relative to control. Concentrations of conventional fungicides were selected according to clinical dosage and standard experimental procedures [30]. PDA was amended with each inhibitor suspension previously sonicated for an hour at room temperature. Conidial suspensions of each strain were prepared by growing the fungi on carboxymethyl cellulose (CMC) medium [31] for 7 days at 25°C and 170 rpm in the dark. Cultures were filtered, the spores were collected by centrifugation and the concentration was adjusted to ~ 10^6^ colony–forming units (CFU)/ml in sterile water. Ten microliters of the conidial suspension of each strain were plated on the amended PDA. Three replicates were prepared for each inhibitor and concentration.

### Measurement of Minimal inhibitory concentration (MIC)

The inhibitory activity of each compound was expressed as Minimal Inhibitory Concentration (MIC) and represents the lowest concentration of active ingredient (μg/ml) that is sufficient to inhibit fungal growth.

### Measurement of Lethal Dose 50 (LD _50_)

The Lethal Dose 50 of each compound was calculated as the concentration of active ingredient (μg/ml) able to reduce by 50% the growth of the fungus *in vitro*.

### Statistical analysis

Data on mycelium weight (mg of dry weight per Petri plate) and mycelium growth (mm per Petri plate), obtained from separate experiments, were expressed as percentage of the relative control treatment and pooled to perform statistical analyses. To compare the activities of different concentrations of each of the selected compounds, a one-way analysis of variance (ANOVA) was performed, followed by multiple comparisons by Tukey HSD test at P = 0.01 using Minitab for Windows, release 17. Prior to performing the ANOVA, normality of residuals was verified by the Shapiro-Wilk test, whereas assumption of homoscedasticity was checked by the Levene test.

## Results

### Antifungal activity of magnolol 1 on a collection of representative isolates of *Fusarium* spp.

The inhibitory effect of magnolol **1** on the dry weight of thirty-two *Fusarium* strains tested in Vogel’s medium is reported on Table 3.

**Table 3:** Mycelium dry weight reduction of 32 *Fusarium* spp. isolates obtained from soil (10 isolates), diseased plants or grain (9 isolates), a water tap and human specimen (13 isolates) in different hospitals treated with magnolol at 0.5 mM.

Species	PVS-Fu n.^a^	Dry weight (% of control)
*F*. *chlamydosporum*	4	30.6 ± 11.4
*F*. *crookwellense*	6	56.5. ± 15.9
*F*. *crookwellense*	406	56.0 ± 10.0
*F*. *guttiforme*	864	0 ± 0
*F*. *langsethiae*	341	18.3 ± 2.4
*F*. *pseudograminearum*	7	28.5 ± 10.8
*F*. *solani*	93	43.6 ± 5.5
*F*. *solani*	97	0 ± 0
*F*. *solani*	100	66.6 ± 29.1
*F*. *solani*	95	64.9 ± 7.3
*F*. *solani*	105	49.0 ± 18.2
*F*. *solani*	107	43.3 ± 12.6
*F*. *solani*	108	34.3 ± 18.2
*F*. *solani*	109	0 ± 0
*F*. *solani*	110	25.7 ± 8.1
*F*. *solani*	111	34.3 ± 18.2
*F*. *subglutinans*	10	8.7 ± 2.1
*F*. *subglutinans*	187	4.1 ± 0.6
*F*. *verticillioides*	400	45.1 ± 22.3
*F*. *verticillioides*	112	8.4 ± 5.0
*F*. *verticillioides*	113	5.2 ± 2.0
*F*. *verticillioides*	88	43.1 ± 10.9
*F*. *oxysporum*	222	60.3 ± 23.9
*F*. *oxysporum*	123	42.9 ± 8.4
*F*. *oxysporum*	124	39.0 ± 3.9
*F*. *oxysporum*	89	22.3 ± 2.6
*F*. *oxysporum*	126	24.2 ± 3.6
*F*. *oxysporum*	127	80.0 ± 17.3
*F*. *oxysporum*	128	49.7 ± 14.7
*F*. *oxysporum*	516	67.1 ± 12.1
*F*. *equiseti*	146	21.5 ± 2.4
*F*. *equiseti*	147	0 ± 0

^*a*^ PVS-Fu n. Collection number of Dipartimento di Agraria, Sezione Patologia Vegetale ed Entomologia, Sassari, Italy.

Except for *F*. *crookwellense* 6, *F*. *crookwellense* 406, *F*. *oxysporum* 127, *F*. *oxysporum* 222, *F*. *oxysporum* 516, *F*. *solani* 95, and *F*. *solani* 100, the remaining 25 *Fusarium* isolates underwent a mycelium dry weight reduction greater than 50% when exposed to magnolol **1** at 0.5 mM (Table 3).

Complete or almost complete inhibition was observed in eight isolates, four of them belonging to clinical *Fusarium* collected from human specimen (PVS-Fu, 864; 95; 112; 113, Table 2 and Table 3). Magnolol **1** at 0.5 mM was very effective on *F*. *guttiforme* 864 cultured from a paronychial infection and against *F*. *verticillioides* 112 and *F*. *verticillioides* 113, collected from pathological human blood, leading to complete inhibition of mycelium growth. Complete inhibition was also observed for *F*. *solani* 97, whereas more than 50% reduction of mycelium dry weight was obtained for *F*. *solani* 105 and *F*. *solani* 93. Similarly, onycomycosis-causing *F*. *oxysporum* 89 and *F*. *oxysporum* 126 were inhibited by over 75%, whereas *F*. *oxysporum* 127 was inhibited only by 20%.

### Selection and activity of two conventional fungicides targeted at *Fusarium* isolates of clinical relevance

Terbinafine and fluconazole, two conventional fungicides, were assayed over a range of 0.1–10 μg/ml and 1–50 μg/ml, respectively, on a set of *F*. *verticillioides*, *F*. *solani* and *F*. *oxysporum* isolates from human samples and clinical environment (Table 2). With different levels of intensity, *F*. *verticillioides* isolates were the most sensitive, whereas *F*. *solani* isolates showed the lowest sensitivity towards both fungicides (Fig A and Fig B in Supporting Information). In particular, *F*. *verticillioides* isolates were highly sensitive to terbinafine, with MICs ranging from 1 to 5 μg/ml and LD_50_ between 0.1 and 1 μg/ml (Table 4).

**Table 4:** LD_50_ and MIC values (in μg/ml) of terbinafine, fluconazole, magnolol 1 and honokiol 2 tested against *Fusarium* spp.

	Terbinafine		Fluconazole		Magnolol 1		Honokiol 2	
	LD_50_	MIC	LD_50_	MIC	LD_50_	MIC	LD_50_	MIC
*F*. *verticillioides* 604	0.1–0.5	1–5	10–25	10–25	10 - <50	>400	5–10	10–100
*F*. *verticillioides* 86	0.5–1	1–5	10–25	10–25	50	>400	5–10	10–100
*F*. *verticillioides* 87	0.5–1	1–5	10–25	10–25	5–10	>400	5	10–100
*F*. *verticillioides* 84	0.1–0.5	1–5	10–25	10–25	50–100	>400	5–10	5–10
*F*. *verticillioides* 83	0.1–0.5	1–5	10–25	10–25	10–50	>400	5–10	10–100
*F*. *verticillioides* 88	0.1–0.5	1–5	10–25	10–25	10–50	>400	5–10	10–100
*F*. *solani* 94	1–5	>10	>50	>50	100	>400	5–10	10–100
*F*. *solani* 96	1–5	>10	>50	>50	10–50	>400	5–10	10–100
*F*. *solani* 502	1–5	>10	>50	>50	5–10	>400	5–10	10–100
*F*. *solani* 93	1–5	>10	>50	>50	10–50	>400	5–10	5–10
*F*. *oxysporum* 89	1–5	5–10	>50	>50	5–10	>400	5–10	10–100
*F*. *oxysporum* 516	1–5	5–10	>50	>50	5–10	>400	5–10	10–100
*F*. *oxysporum* 92	1–5	1–5	>50	>50	5–10	>400	5–10	10–100
*F*. *oxysporum* 90	1–5	5–10	>50	>50	5–10	>400	5–10	10–100
*F*. *oxysporum* 91	1–5	5–10	>50	>50	5–10	>400	<5	10 -<100

At 25 μg/ml, fluconazole was able to completely inhibit the vegetative growth of all *F*. *verticillioides* isolates, whereas almost 60% mycelium growth inhibition was observed for *F*. *solani* 502 and for all *F*. *oxysporum* isolates. The mycelium growth of *F*. *solani* 94 and *F*. *solani* 96 was not affected by fluconazole even at the highest concentration of 50 μg/ml. Even at lower concentrations (5–10 μg/ml), terbinafine showed more effective antifungal activity (growth inhibition being > 50%) than fluconazole in all isolates belonging to the three *Fusarium* species complexes (Fig A and Fig B in Supporting Information).

### Antifungal activity of magnolol 1, honokiol 2 and their derivatives 3–8

Isolates belonging to *F*. *oxysporum* were more sensitive to magnolol **1** compared to the other species (Table 4). Magnolol **1** affected all isolates belonging to the three species complexes with a mycelium growth inhibition >50% at 100 μg/ml; the trend was not significantly different at 200 μg/ml and for *F*. *verticillioides* 604, *F*. *solani* 502, *F*. *oxysporum* 89, *F*. *oxysporum* 90 and *F*. *oxysporum* 91 even at 400 μg/ml (Fig C in Supporting Information). MIC of magnolol **1** was >400 μg/ml for all the tested *Fusarium* isolates (Table 4).

Over a concentration range of 10–100 μg/ml, honokiol **2** completely inhibited mycelium growth in all 15 fungal strains investigated (Fig D in Supporting Information). More than 50% mycelium growth inhibition was observed in all *Fusarium* spp. when honokiol **2** was amended at 10 μg/ml. In *F*. *verticillioides* 84, a fungus responsible for chronic sinusitis, honokiol **2** showed strong antifungal activity with MIC between 5 and 10 μg/ml (Table 4). Honokiol **2** was more effective than magnolol **1** at inhibiting *F*. *solani*, with MIC ranging from 10 to 100 μg/ml, except for *F*. *solani* 93, whose MIC was comprised between 5 and 10 μg/ml. Besides *F*. *oxysporum* 91, antifungal activity of magnolol **1** was comparable to that of honokiol **2** in *F*. *oxysporum* isolates, with a LD_50_ ranging from 5 to 10 μg/ml. However, magnolol **1** could not completely inhibit fungal growth at the tested concentrations, whereas honokiol **1** did over a range of 10–100 μg/ml.

A mono protection of the phenol-OH with an acetyl group in magnolol **1** (compound **3**) had a higher effect on MIC values in *F*. *verticillioides* 83, *F*. *verticillioides* 86, *F*. *verticillioides* 88 and *F*. *solani* 93 (Table 5 and Fig E in Supporting Information).

**Table 5:** LD_50_ and MIC values (in μg/ml) of magnolol and honokiol derivatives 3–8 tested against *Fusarium* spp.

	Magnolol monoacetate 3		Magnolol diacetate 4		Magnolol mono glucopyranoside 8		Honokiol 2-monoacetate 5		Honokiol 4ʹ-monoacetate 6		Honokiol diacetate 7	
	LD_50_	MIC	LD_50_	MIC	LD_50_	MIC	LD_50_	MIC	LD_50_	MIC	LD_50_	MIC
*F*. *verticillioides* 604	10–50	>400	>400	>400	>400	>400	50–100	>100	>100	>100	>100	>100
*F*. *verticillioides* 86	10–50	200–400	>400	>400	>400	>400	50–100	>100	25–50	>100	>100	>100
*F*. *verticillioides* 87	10–50	>400	>400	>400	>400	>400	50–100	>100	25–50	>100	>100	>100
*F*. *verticillioides* 84	10–50	>400	>400	>400	>400	>400	50–100	>100	25–50	>100	>100	>100
*F*. *verticillioides* 83	10–50	200–400	>400	>400	>400	>400	25–50	>100	25–50	>100	>100	>100
*F*. *verticillioides* 88	10–50	100–400	>400	>400	>400	>400	25–50	>100	25–50	>100	>100	>100
*F*. *solani* 94	50–100	>400	>400	>400	>400	>400	>100	>100	>100	>100	>100	>100
*F*. *solani* 96	100–200	>400	>400	>400	>400	>400	>100	>100	>100	>100	>100	>100
*F*. *solani* 502	10–50	>400	>400	>400	>400	>400	50–100	>100	25–50	>100	>100	>100
*F*. *solani* 93	50–100	200–400	>400	>400	>400	>400	>100	>100	>100	>100	>100	>100
*F*. *oxysporum* 89	10–50	>400	>400	>400	>400	>400	25–50	>100	25–50	>100	>100	>100
*F*. *oxysporum* 516	10–50	>400	>400	>400	>400	>400	50–100	>100	25–50	>100	>100	>100
*F*. *oxysporum* 92	10–50	>400	>400	>400	>400	>400	25–50	>100	25–50	>100	>100	>100
*F*. *oxysporum* 90	10–50	>400	>400	>400	>400	>400	25–50	>100	25–50	>100	>100	>100
*F*. *oxysporum* 91	10–50	>400	>400	>400	>400	>400	25–50	>100	25–50	>100	>100	>100

At 400 μg/ml, almost complete growth inhibition was observed in *F*. *oxysporum* 89, *F*. *solani* 93, *F*. *verticillioides* 83, *F*. *verticillioides* 84, *F*. *verticillioides* 86, and *F*. *verticillioides* 88. At 100 μg/ml, magnolol mono acetate **3** inhibited by 50% the mycelium growth of all the tested strains (Fig E in Supporting Information). Conversely, magnolol mono glucopyranoside **8** was not effective at concentration up to 400 μg/ml on all the *Fusarium* species complexes (Fig K in Supporting Information).

Honokiol mono acetate **5** and **6** proved less effective than the parent compound, with LD_50_ and MIC values higher than those displayed by honokiol **2** (Table 5). At 100 μg/ml, except for *F*. *solani* 94, *F*. *solani* 96 and *F*. *solani* 93, honokiol 2-mono acetate **5** and honokiol 4ʹ-mono acetate **6** inhibited by 50% the vegetative growth of all *Fusarium* isolates (Fig G and Fig H in Supporting Information). The colony growth of *F*. *verticillioides* 87, *F*. *verticillioides* 88 and *F*. *oxysporum* 90 was inhibited by 80% in the presence of honokiol 2-mono acetate **5** at 100 μg/ml. Honokiol diacetate **7** was less effective against all the *Fusarium* species complexes (Table 5 and Fig I in Supporting Information). Complete protection of both phenol-OH groups in magnolol **1** and honokiol **2** determined a reduction of their efficacy (Table 5 and Fig F and Fig I in Supporting Information).

Compounds **1**–**7** showed comparable lipophilicity estimated by LogP between 4.99 and 5.03, while LogP 2.36 was calculated for magnolol mono glucopyranoside **8**.

### Comparison of antifungal activity of compounds 1–8 with the conventional fungicides

When the activity of magnolol **1**, honokiol **2** and their derivatives **3**–**8** was compared with that of terbinafine and fluconazole, a different trend of efficacy was observed (Table 4 and Table 5 and Figs A-K in Supporting Information).

**Fig 2:**
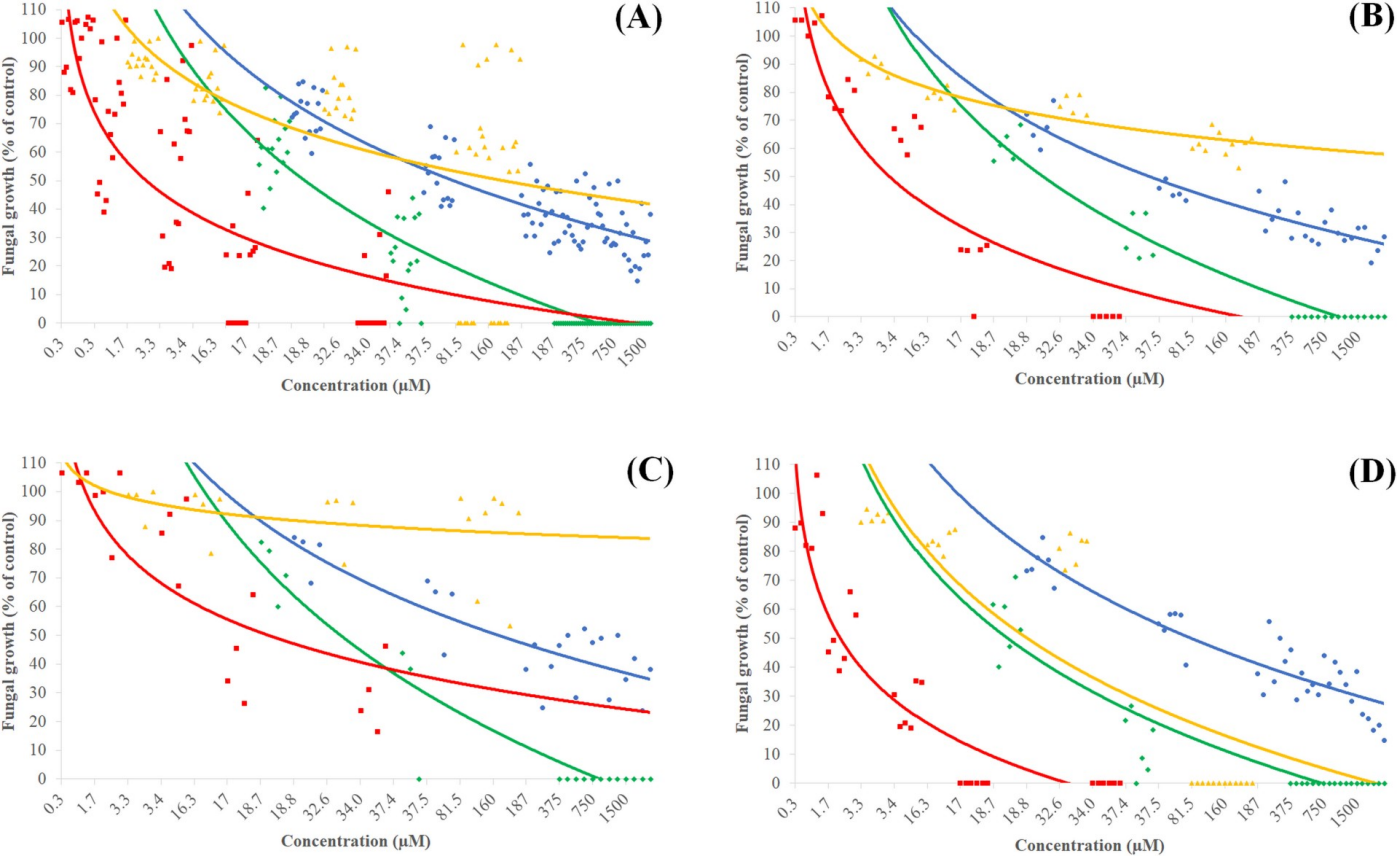
The effect of different molarity solutions of terbinafine (red), fluconazole (yellow), magnolol **1** (blue), and honokiol **2** (green) on fungal growth of: (A) *Fusarium oxysporum*, *F*. *solani* and *F*. *verticillioides*; (B) *F*. *oxysporum*; (C) *F*. *solani* and (D) *F*. *verticillioides*.

With the exception of *F*. *solani* 94, LD_50_ of magnolol **1** for both *F*. *solani* and *F*. *oxysporum* species complexes was lower than that of fluconazole. Magnolol **1** showed better or similar inhibitory activity than fluconazole at 5 μg/ml, whereas honokiol **2** showed a higher inhibition of mycelium growth compared to the conventional fungicide for all clinical *Fusarium* species complexes (Figs B-D in Supporting Information). Conversely to terbinafine and fluconazole, honokiol **2** showed comparable inhibitory activity against all three species complexes (Fig D in Supporting Information). Table A in Supporting Information reports a comparison of μg/ml and molarity for all investigated compounds with that of fluconazole and terbinafine. Furthermore, the growth inhibition data of terbinafine, fluconazole, magnolol **1** and honokiol **2** were collected in four graphics where concentrations in μg/ml were converted to molarity (Fig 2A, 2B, 2C and 2D). In Fig 2A the summary effect of terbinafine, fluconazole, magnolol **1** and honokiol **2** on *F*. *oxysporum*, *F*. *solani* and *F*. *verticillioides* is shown. Terbinafine and honokiol **2** were both able to inhibit mycelium growth effectively. Nevertheless, terbinafine, at the tested clinical dosages, could not completely inhibit fungal growth as honokiol **2** did on all *Fusarium* isolates over a range of 37.5–375 μM (10–100 μg/ml). The effect of magnolol **1** and fluconazole is superimposable over a range of concentrations, roughly between 19 and 160 μM, though magnolol **1** was more effective as vegetative growth inhibitor.

Comparable LD_50_ was observed on *F*. *oxysporum* 91 collected from onychomycosis, when treated with terbinafine (1–5 μg/ml, 3.4–17 μM) and honokiol **2** (<5 μg/ml, < 18.8 μM) (Table 4). At clinical dosages, fluconazole was not effective against this set of *F*. *oxysporum* isolates (Fig 2B), whereas magnolol **1** and honokiol **2** were more effective, the latter being able to provide complete fungal growth inhibition at 0.375 mM (100 μg/ml).

Except for *F*. *solani* isolates, terbinafine was able to inhibit mycelium growth over a range between 3.4–34.3 μM (10–100 μg/ml), i.e., smaller by roughly one order of magnitude compared to honokiol **2** (Fig 2C). Over a range of 5–10 μg/ml (18.8–37.5 μM), honokiol **2** was as effective as terbinafine (10 μg/ml, 34 μM) in inhibiting the colony growth (MIC) of *F*. *solani* 93, isolated from foot dermatomycosis (Table 3). Moreover, at 10 μg/ml, honokiol **2** (37.5 μM) had a similar effect compared to that of terbinafine (34 μM) in inhibiting *F*. *solani* 502 isolated from a hospital basin sink and, to a lesser extent, *F*. *oxysporum* 516 collected from a hospital water tap (Table 4 and Fig A and Fig D in Supporting Information). Terbinafine and fluconazole were able to inhibit fungal growth of *F*. *verticillioides* with MIC value < 5 μg/ml and < 25 μg/ml, respectively (Table 4), while honokiol **2** followed the same trend as terbinafine, providing a complete fungal growth inhibition at <100 μg/ml.

## Discussion

The objective of this work was to propose ‘natural’ alternatives to the use of conventional fungicides, even if this approach required the use of higher concentrations of the identified compounds. The compounds we tested, namely magnolol **1** and honokiol **2**, are commercially available in large amounts and have been used in modern clinical practice in Eastern Asia, while in the United States and Europe are considered cosmetic additives.

In a preliminary screening, the selection of *Fusarium* spp. from agricultural and human sources provided a general information of the potential antifungal activity of magnolol **1** towards these fungi, including ubiquitous representatives of the *F*. *solani* and *F*. *oxysporum* species complexes. With different ranges of intensity, magnolol **1** was able to control all *F*. *oxysporum* and *F*. *solani* isolates sampled from human specimen. Magnolol **1** also proved a potential candidate to control *F*. *subglutinans* and, to a lesser extent, *F*. *verticillioides* 400, two species able to infect corn. Data reported in Table 3 highlight a different sensitivity of isolates within the same species complex, regardless of their origin.

Subsequently, this study was carried out on *Fusarium* spp. of clinical interest, aiming at testing the antifungal activity of magnolol **1**, honokiol **2** and their derivatives **3**–**8** as sustainable alternatives to current clinical drug treatments, which suffer from frequent emergence of resistant pathogen populations. Two commercial fungicides with different mode of action, i.e. terbinafine and fluconazole, were selected in order to compare their antifungal activity with that of compounds **1**–**8** against all clinical *Fusarium* spp. isolates. Terbinafine, a tertiary allylamine and one of the most effective antimycotic drugs used in human therapy, inhibits the ergosterol biosynthesis by acting on the squalene epoxidation and is widely used for the treatment of nail infections [32]. Fluconazole, an inhibitor of the lanosterol 14α-demethylase, belongs to the azole SBI class I, whose activity is weaker than that of terbinafine. Although acquired resistance against fluconazole was observed in *Fusarium* spp. of agricultural interest, the fungicide is still being used in the clinical treatment of these fungi [33, 34]. Concentrations of terbinafine and fluconazole were selected according to literature [30].

Magnolol **1**, honokiol **2** and their derivatives **3**–**8** were tested at concentrations ranging from twenty-five-fold lower and, roughly three-fold higher than those used to amend Vogel’s medium in the preliminary assay. This concentration range allowed us to compare the species diversity and the antifungal susceptibility profile of the clinical isolates with all tested. Over an acceptable range of variability, percentage of growth inhibition of *F*. *verticillioides* 88, *F*. *solani* 93, *F*. *oxysporum* 89 and *F*. *oxysporum* 516 observed in Vogel’s assay with magnolol **1** was comparable to that observed on PDA, suggesting that growth inhibition is independent from the cultural medium. In our screening, magnolol **1** and its structural isomer, honokiol **2,** showed effective antifungal activity. These compounds may prove useful in antimicrobial drug development, based on their recognised multitarget bioactivities, market availability and almost safe metabolic profile in human body [25, 26]. Moreover, the capacity of magnolol **1** and honokiol **2** to act on multiple cell targets can be the key to control infection by resistant fungi which do not respond to conventional therapies.

When the concentrations applied were analysed in terms of molarity, a clearer comparison of the antifungal activities of terbinafine, fluconazole, magnolol **1** and honokiol **2** was obtained. Even though different ranges of molarity were taken into account, a very similar trend in the activities of terbinafine and honokiol **2** was observed. In the case of *F*. *oxysporum* 91, honokiol **2** was very effective, thereby appearing as a promising antifungal agent in the treatment of onychomycoses. Interestingly, the antifungal activity of honokiol **2** towards *F*. *solani* 93, isolated from foot dermatomycosis, and, to a lesser extent, against *F*. *solani* 502, isolated from hospital basin sink, was comparable to that exerted by terbinafine. Due to the acceptable metabolic profile of honokiol **2** taken in oral dosage, these results suggest a potential application of this compound in washing water contaminated by *F*. *oxysporum* and in the sanitation of sinks.

A pro-drug approach [29] was applied as a versatile strategy to improve the bioactivity of these compounds by transformation of the hydroxyl groups of magnolol **1** and honokiol **2** in a mono- and di-acetyl ester, respectively, and, in the case of magnolol **1**, in a mono glucosyl acetal **8**. The antifungal activity of honokiol **2** dropped when one of the phenol-OH was protected with an acetyl group (i.e. in compounds **5** and **6**). The effect was less remarkable between honokiol 4ʹ-mono acetate **6** and honokiol 2-mono acetate **5**, further highlighting the importance of both phenol-OH groups in the activity of honokiol **2**. On the contrary, magnolol mono acetate **3** showed higher inhibitory activity than the corresponding parental compound, whereas the bioactivity dropped when phenol-OH groups were protected with two acetyl groups.

Due to the presence of a glucosyl unit, magnolol mono glucopyranoside **8** was more hydrophilic compared to compounds **1**–**7**. Although a free phenol-OH group is present in the structure of this compound, it is possible that its poor antifungal activity is a result of its failure to reach its target in the fungal cell through the lipophilic membrane.

Magnolol **1** and honokiol **2** are structural isomers, but the different position of one phenol-OH group confers distinct conformations and electronic effects and, as a result, a different reactivity (e.g., antioxidant activity) [7]. A C_2_-symmetry axis in magnolol **1** allows only one monoester isomer, whereas two distinct isomers can be produced after monoesterification of honokiol **2**. Phenol-OH groups in magnolol **1** have different acidity due to the formation of an intramolecular H-bond between the two phenol-OH. On the contrary, in honokiol **2**, the large dihedral angle and the distance of the two phenol-OH groups reduce the conjugation effect. These features reflect the different activity that monoesters and diesters of magnolol **1** and honokiol **2** have displayed during *in vitro* assays. According to the position of the two phenol-OH groups in the biphenyl structure, a different mechanism of action between magnolol **1** and honokiol **2** might take place when these compounds interact with fungi.

Several modes of action of magnolol **1** and honokiol **2** were described towards fungi [35–37]. Magnolol **1** interacts with ergosterol present in the cell membrane, inducing a partial disruption of the structure, but its mechanism of action is likely to differ from that of other inhibitors of sterol biosynthesis as it has proven effective also on fluconazole-resistant *Candida* spp. isolates [38]. Cell wall components such as β-1,3-glucans were proposed as potential target of magnolol, similarly to fungicides of the echinocandin family [39].

It is reasonable to hypothesize that other mechanisms underlay the antifungal activity of magnolol **1** and honokiol **2** that may justify a differential action towards the three investigated *Fusarium* species complexes. Recently, the role of honokiol **2** as activator of mitochondrial ROS by mitochondrial dysfunction and depolarisation of mitochondrial membrane potential in *C*. *albicans* was highlighted [40]. Moreover, honokiol **2** is able to hinder the high content of pro-oxidant iron ions in fungi by sequestering the ion [41]. Having different targets and multiple mechanisms of action, it is likely that honokiol **2** acts more effectively than magnolol **1** on selected fungal targets. In fact, candidates that are able to induce dysfunction of mitochondrial membrane and alteration of iron homeostasis represent thenew pharmacological leads currently under development [42]. The scarce toxicity of magnolol **1** upon dermal and oral administration, which is even lower than that of honokiol **2** [25, 26], opens new straightforward clinical applications for this compound.

In conclusion, our findings suggest that magnolol **1**, honokiol **2** and magnolol acetate **3** may represent promising alternatives for the treatment of fungal infections on both plants and humans: they share a different mode of action compared to conventional antifungal drugs, whose clinical application is jeopardized by the onset of resistance phenomena in fungal populations. The low toxicity of magnolol **1** and honokiol **2**, their low cost and their efficacy against both yeast and filamentous fungi prompt further investigation on other fungal pathogens relevant in human or plant clinics and, to a large extent, on fungal pathogens of ecological concern.

## Supporting information

S1 Text Supplementary material and methods, synthesis and references. Comparison of μg/ml and molarity (μM or mM) for compounds 3–8 with that of fluconazole and terbinafine (Table A).
Raw datasets of the effect of compounds 1–8, terbinafine and fluconazole on mycelium growth of *Fusarium oxysporum*, *Fusarium verticillioides* and *Fusarium solani* (Table B).
Antifungal activity of terbinafine (Fig A). Antifungal activity of fluconazole (Fig B).Antifungal activity of compound 1 (Fig C). Antifungal activity of compound 2 (Fig D). Antifungal activity of compound 3 (Fig E). Antifungal activity of compound 4 (Fig F). Antifungal activity of compound 5 (Fig G). Antifungal activity of compound 6 (Fig H). Antifungal activity of compound 7 (Fig I). Antifungal activity of compound 8 (Fig K). Photos of mycelium growth of five *Fusarium oxysporum* isolates in the presence of magnolol 1 at 5 and 400 μg/ml in comparison with control (Fig L).
Photos of mycelium growth of six *Fusarium verticillioides* isolates in the presence of magnolol 1 at 5 and 400 μg/ml in comparison with control (Fig M).
Photos of mycelium growth of four *Fusarium solani* isolates in the presence of magnolol 1 at 5 and 400 μg/ml in comparison with control (Fig N).
(ZIP)
