# Supplementary material for: Honokiol, magnolol and its monoacetyl derivative show strong anti-fungal effect on Fusarium isolates of clinical relevance
Source: PLoS One. 2019 Sep 4;14(9):e0221249. doi: 10.1371/journal.pone.0221249 (PMC6726233; doi:10.1371/journal.pone.0221249)
Supplement: S1 Text — Comparison of μg/ml and molarity (μM or mM) for compounds 3–8 with that of fluconazole and terbinafine (Table A). Raw datasets of the effect of compounds 1–8, terbinafine and fluconazole on mycelium growth of Fusarium oxysporum, Fusarium verticillioides and Fusarium solani (Table B). Antifungal activity of terbinafine (Fig A). Antifungal activity of fluconazole (Fig B).Antifungal activity of compound 1 (Fig C). Antifungal activity of compound 2 (Fig D). Antifungal activity of compound 3 (Fig E). Antifungal activity of compound 4 (Fig F). Antifungal activity of compound 5 (Fig G). Antifungal activity of compound 6 (Fig H). Antifungal activity of compound 7 (Fig I). Antifungal activity of compound 8 (Fig K). Photos of mycelium growth of five Fusarium oxysporum isolates in the presence of magnolol 1 at 5 and 400 μg/ml in comparison with control (Fig L). Photos of mycelium growth of six Fusarium verticillioides isolates in the presence of magnolol 1 at 5 and 400 μg/ml in comparison with control (Fig M). Photos of mycelium growth of four Fusarium solani isolates in the presence of magnolol 1 at 5 and 400 μg/ml in comparison with control (Fig N). (ZIP) [file pone.0221249.s001.zip › A_Table.docx]

**Table A**

| compound | Concentration range in µg/mL (ppm) | Molarity range | Concentration in µg/mL(ppm) and molarity used in each assay |
| --- | --- | --- | --- |
| Terbinafine | 0.1 – 10 µg/mL | 0.34 µM – 34.3 µM | 0.1 µg/mL (0.34 µM),  0.5 µg/mL (1.7 µM),  1. µg/mL (3.4 µM),  5 µg/mL (17 µM),  10 µg/mL (34 µM) |
| Fluconazole | 1-50 µg/mL | 3.26 µM - 0.16 mM | 1.0 µg/mL (3.26 µM),  5 µg/mL (16.3 µM),  10 µg/mL (32.6 µM),  25 µg/mL (81.5 µM),  50 µg/mL (0.16 mM) |
| Magnolol **1** | 5 – 400 µg/mL | 18.8 µM - 1.50 mM | 5 µg/mL (18.8 µM),  10 µg/mL (37.5 µM),  50 µg/mL (0.187 mM),  100 µg/mL (0.375 mM),  200 µg/mL (0.75 mM),  400 µg/mL (1.50 mM) |
| Honokiol **2** | 5 – 400 µg/mL | 18.8 µM - 1.50 mM | 5 µg/mL (18.8 µM),  10 µg/mL (37.5 µM),  100 µg/mL (0.375 mM),  200 µg/mL (0.75 mM)  400 µg/mL (1.50 mM) |
| Magnolol monoacetate **3** | 5 – 400 µg/mL | 16.23 µM - 1.32 mM | 5 µg/mL (16.2 µM),  10 µg/mL (32.5 µM),  50 µg/mL (0.162 mM),  100 µg/mL (0.324 mM),  200 µg/mL (0.649 mM),  400 µg/mL (1.32 mM) |
| Magnolol diacetate **4** | 50 – 400 µg/mL | 0.14 mM - 1.1 mM | 50 µg/mL (0.14 µM),  100 µg/mL (0.28 mM),  200 µg/mL (0.57 mM),  400 µg/mL (1.14 mM) |
| Honokiol monoacetate **5** | 5 – 100 µg/mL | 16.23 µM - 0.32 mM | 5 µg/mL (16.2 µM),  10 µg/mL (32.5 µM),  25 µg/mL (81.15 µM),  50 µg/mL (0.16 mM),  100 µg/mL (0.32 mM) |
| Honokiol monoacetate **6** | 5 – 100 µg/mL | 16.23 µM - 0.32 mM | 5 µg/mL (16.23 µM),  10 µg/mL (32.46 µM),  25 µg/mL (81.15 µM),  50 µg/mL (0.16 mM),  100 µg/mL (0.32 mM) |
| Honokiol diacetate **7** | 5 – 100 µg/mL | 14.28 µM - 0.28 mM | 5 µg/mL (14.28 µM),  10 µg/mL (28.56 µM),  25 µg/mL (71.4 µM),  50 µg/mL (0.14 mM),  100 µg/mL 0.285 mM) |
| Magnolol mono glucopyranoside **8** | 5 – 400 µg/mL | 11.68 µM - 0.93 mM | 5 µg/mL (11.68 µM),  10 µg/mL (23.36 µM),  100 µg/mL (0.23 mM),  200 µg/mL (0.47 mM),  400 µg/mL (0.93 mM) |
