# Supplementary material for: Honokiol, magnolol and its monoacetyl derivative show strong anti-fungal effect on Fusarium isolates of clinical relevance
Source: PLoS One. 2019 Sep 4;14(9):e0221249. doi: 10.1371/journal.pone.0221249 (PMC6726233; doi:10.1371/journal.pone.0221249)
Supplement: S1 Text — Comparison of μg/ml and molarity (μM or mM) for compounds 3–8 with that of fluconazole and terbinafine (Table A). Raw datasets of the effect of compounds 1–8, terbinafine and fluconazole on mycelium growth of Fusarium oxysporum, Fusarium verticillioides and Fusarium solani (Table B). Antifungal activity of terbinafine (Fig A). Antifungal activity of fluconazole (Fig B).Antifungal activity of compound 1 (Fig C). Antifungal activity of compound 2 (Fig D). Antifungal activity of compound 3 (Fig E). Antifungal activity of compound 4 (Fig F). Antifungal activity of compound 5 (Fig G). Antifungal activity of compound 6 (Fig H). Antifungal activity of compound 7 (Fig I). Antifungal activity of compound 8 (Fig K). Photos of mycelium growth of five Fusarium oxysporum isolates in the presence of magnolol 1 at 5 and 400 μg/ml in comparison with control (Fig L). Photos of mycelium growth of six Fusarium verticillioides isolates in the presence of magnolol 1 at 5 and 400 μg/ml in comparison with control (Fig M). Photos of mycelium growth of four Fusarium solani isolates in the presence of magnolol 1 at 5 and 400 μg/ml in comparison with control (Fig N). (ZIP) [file pone.0221249.s001.zip › G_Fig.pptx]

## Slide 1
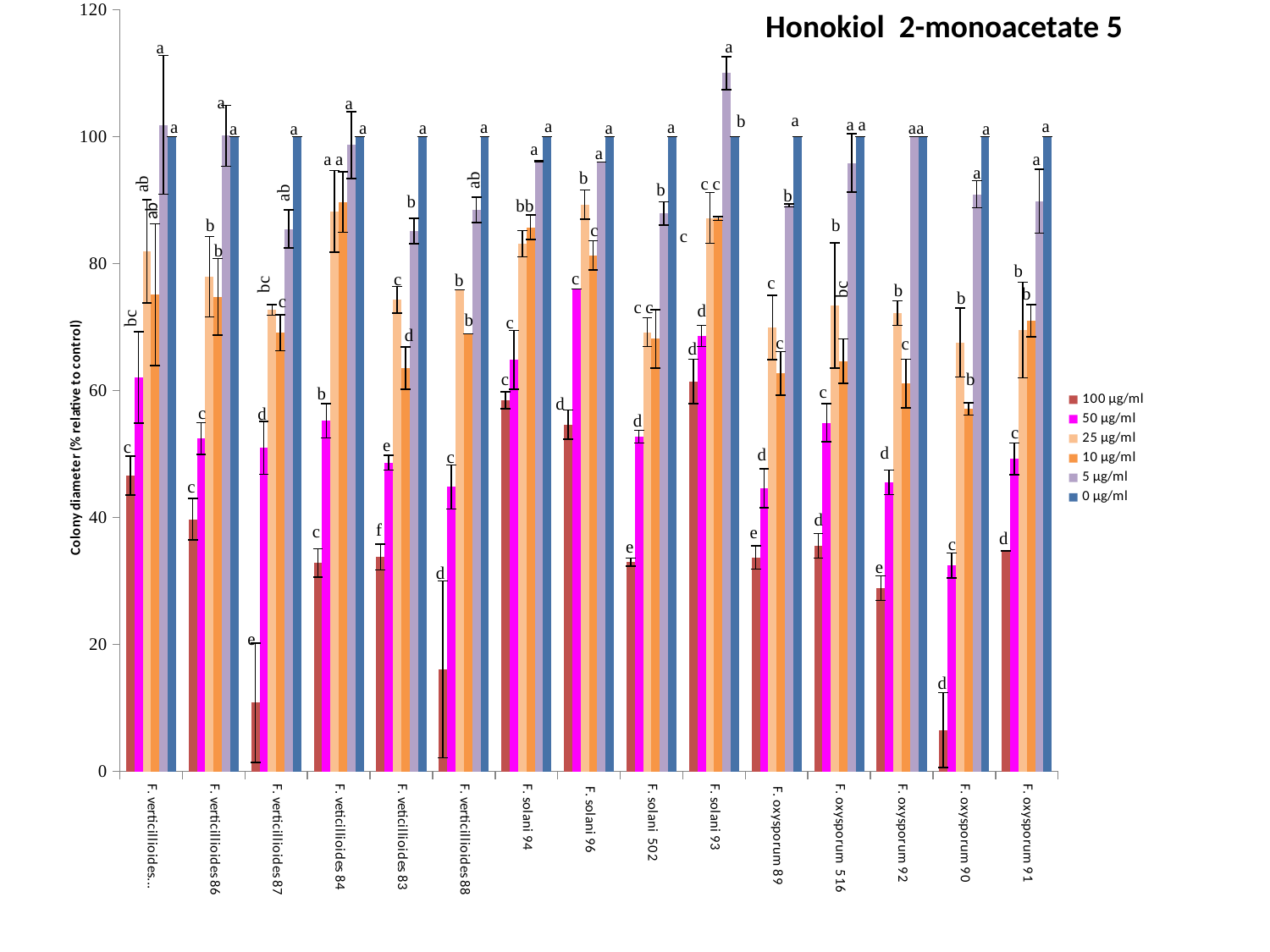

### Chart
| Category | 100 µg/ml | 50 µg/ml | 25 µg/ml | 10 µg/ml | 5 µg/ml | 0 µg/ml |
|---|---|---|---|---|---|---|
| F. verticillioides 604 | 46.611111111111114 | 62.11616161616163 | 81.96969696969697 | 75.12121212121212 | 101.87878787878788 | 100.0 |
| F. verticillioides 86 | 39.74025974025974 | 52.46031746031747 | 77.97258297258297 | 74.7907647907648 | 100.15151515151514 | 100.0 |
| F. verticillioides 87 | 10.818713450292398 | 50.97465886939572 | 72.70955165692008 | 69.10331384015593 | 85.47758284600388 | 100.0 |
| F. veticillioides 84 | 32.85024154589372 | 55.27950310559007 | 88.26777087646654 | 89.71704623878537 | 98.68875086266392 | 100.0 |
| F. veticillioides 83 | 33.77777777777778 | 48.666666666666664 | 74.33333333333333 | 63.555555555555564 | 85.16666666666667 | 100.0 |
| F. verticillioides 88 | 16.091954022988507 | 44.82758620689655 | 75.86206896551725 | 68.96551724137932 | 88.50574712643679 | 100.0 |
| F. solani 94 | 58.46153846153846 | 64.87179487179488 | 83.12820512820512 | 85.74358974358977 | 96.10256410256409 | 100.0 |
| F. solani 96 | 54.666666666666664 | 76.0 | 89.33333333333333 | 81.33333333333333 | 96.0 | 100.0 |
| F. solani 502 | 32.97491039426524 | 52.75985663082437 | 69.21146953405018 | 68.1720430107527 | 87.92114695340501 | 100.0 |
| F. solani 93 | 61.47342995169083 | 68.59903381642512 | 87.19806763285025 | 87.1376811594203 | 110.024154589372 | 100.0 |
| F. oxysporum 89 | 33.730158730158735 | 44.620811287477956 | 69.9294532627866 | 62.6984126984127 | 89.15343915343915 | 100.0 |
| F. oxysporum 516 | 35.555555555555564 | 54.94949494949495 | 73.43434343434343 | 64.64646464646465 | 95.85858585858587 | 100.0 |
| F. oxysporum 92 | 28.88888888888889 | 45.55555555555555 | 72.22222222222223 | 61.111111111111114 | 100.0 | 100.0 |
| F. oxysporum 90 | 6.512820512820514 | 32.46153846153846 | 67.58974358974359 | 57.12820512820513 | 90.9230769230769 | 100.0 |
| F. oxysporum 91 | 34.78260869565218 | 49.275362318840585 | 69.56521739130436 | 71.0144927536232 | 89.85507246376812 | 100.0 | Honokiol 2-monoacetate 5
