# Supplementary material for: Honokiol, magnolol and its monoacetyl derivative show strong anti-fungal effect on Fusarium isolates of clinical relevance
Source: PLoS One. 2019 Sep 4;14(9):e0221249. doi: 10.1371/journal.pone.0221249 (PMC6726233; doi:10.1371/journal.pone.0221249)
Supplement: S1 Text — Comparison of μg/ml and molarity (μM or mM) for compounds 3–8 with that of fluconazole and terbinafine (Table A). Raw datasets of the effect of compounds 1–8, terbinafine and fluconazole on mycelium growth of Fusarium oxysporum, Fusarium verticillioides and Fusarium solani (Table B). Antifungal activity of terbinafine (Fig A). Antifungal activity of fluconazole (Fig B).Antifungal activity of compound 1 (Fig C). Antifungal activity of compound 2 (Fig D). Antifungal activity of compound 3 (Fig E). Antifungal activity of compound 4 (Fig F). Antifungal activity of compound 5 (Fig G). Antifungal activity of compound 6 (Fig H). Antifungal activity of compound 7 (Fig I). Antifungal activity of compound 8 (Fig K). Photos of mycelium growth of five Fusarium oxysporum isolates in the presence of magnolol 1 at 5 and 400 μg/ml in comparison with control (Fig L). Photos of mycelium growth of six Fusarium verticillioides isolates in the presence of magnolol 1 at 5 and 400 μg/ml in comparison with control (Fig M). Photos of mycelium growth of four Fusarium solani isolates in the presence of magnolol 1 at 5 and 400 μg/ml in comparison with control (Fig N). (ZIP) [file pone.0221249.s001.zip › S1_Text.docx]

**S1 Text**

**Material and Methods**

Magnolol **1** and honokiol **2** were purchased from Chemos GmbH, Germany. All the other reagents were of commercial quality and used as purchased from various producers (Sigma-Aldrich, Merck). Flash chromatography was carried out with silica gel 60 (230±400 mesh, Kiesgel, EM Reagents) eluting with appropriate solution in the stated v: v proportions. Analytical thin-layer chromatography (TLC) was performed with 0.25 mm thick silica gel plates (Polygram1 Sil G/UV254, Macherey-Nagel). Solvents were used without additional purification or drying, unless otherwise noted.

^1^H NMR and ^13^C NMR spectra were recorded on spectrometer Varian Mercury Plus operating at 399.93 MHz and 100.57 MHz, respectively. Chemical shifts are given in ppm (δ) and coupling constants in Hertz (Hz); multiplicities are indicated by s (singlet), d (doublet), dd (doublet of doublets), m (multiplet), series of m (series of multiplet) and br (broad signal). CDCl_3_, acetone-*d6*, DMSO-*d6* were used as solvents as indicated below.

Structure of compound **8** was confirmed by comparison with spectroscopic data present in literature of an identical β-anomer achieved under enzymatic conditions [S1].

**Synthesis**

**Compound 3.** To a solution of magnolol **1** (1 g, 3.8 mmol) in dry acetone (10 mL) potassium

carbonate (0.5 g, 3.8 mmol) was added under N_2_. The reaction mixture was stirred at rt for 10 min. Acetic anhydride (0.35 mL, 3.8 mmol) was added and, after stirring at rt for 1 h, the solution was filtered and rotoevaporated to obtain a viscous oil that was purified by flash chromatography using a dichloromethane as eluent.

**Compound 3** [S2]: (oil) (0.95 g 81%): ^1^H NMR (CDCl_3_) δ 1.98 (s, 3H), 3.29 (d, *J* = 7.2 Hz, 2H), 3.40 (d, *J* = 7.2 Hz, 2H), 5.02-5.16 (series of m, 4H), 5.99 (m, 2H), 6.92 (d, *J* = 8.4 Hz, 1H), 6.93 (d, *J* = 2.4 Hz, 1H), 7.08 (dd, *J* = 2.4, 8.0 Hz, 1H), 7.14 (d, *J* = 8.4 Hz, 1H), 7.21 (d, *J* = 2.4 Hz, 1H), 7.24 (dd, *J* = 2.4, 8.0 Hz, 1H); ^13^C NMR (CDCl_3_) δ 20.61, 39.32, 39.57, 115.58, 116.29, 116.45, 122.91, 123.92, 129.55, 129.71, 130.11, 130.63, 131.85, 131.94, 136.77, 137.79, 138.71, 146.84, 151.30, 169.91; Anal. Calcd for C_20_H_20_O_3_: C, 77.90; H, 6.54; Found: C, 78.05; H, 6.58.

**Compound 4**. To a solution of magnolol **1** (1 g, 3.8 mmol) in dry acetone (10 mL) potassium carbonate (1.03 g, 7.5 mmol) was added under N_2_. The reaction mixture was stirred at rt for 10 min. Acetic anhydride (0.8 mL, 8.2 mmol) was added and, after stirring at rt for 1 h, the solution was filtered and rotoevaporated to obtain a viscous oil that was purified by flash chromatography using a dichloromethane as eluent.

**Compound 4** [S2]: (oil) (0.98 g, 74%): ^1^H NMR (CDCl_3_) δ 1.96 (s, 6H), 3.30 (d, *J* = 6.4 Hz, 4H), 5.07 (m, 4H), 5.79 (m, 2H), 6.92 (d, *J* = 8.4 Hz, 2H), 7.03 (d, *J* = 2.0 Hz, 2H), 7.47 (dd, *J* = 2.0, 8.4 Hz, 2H); ^13^C NMR (CDCl_3_) δ 21.74, 39.50, 116.20, 122.01, 129.82, 130.24, 131.23, 135.98, 137.68, 145.30, 169.46; C_22_H_22_O_4_: C, 75.41; H, 6.33; Found: C, 76.45; H, 5.87.

**Compounds 5, 6.** To a solution of honokiol **2** (1 g, 3.76 mmol) in dry acetone (20 mL) potassium carbonate (0.57 g, 3.76 mmol) was added at 0 °C under N_2_. The reaction mixture was stirred at rt for 30 min. Acetic anhydride (0.42 mL g, 4.13 mmol) was added and, after stirring at rt for 1 h, the solution was filtered and rotoevaporated to obtain a viscous oil that was purified by flash chromatography using a petroleum ether: tetrahydrofuran 10:2 solution as eluent to obtain: **compound 5** (oil) (0.11 g, 10 %), **compound 6** (oil) (0.44 g, 38%), **compound 7** (oil) (0.24 g, 18%) and starting material (honokiol **2**) (0.2 g, 20%).

**Compound 5**: ^1^H NMR (CDCl_3_) δ 2.07 (s, 3H), 3.40 (m, 4H), 5.04-5.19 (series of m, 4H), 5.69 (bs, 1H), 6.01 (m, 2H), 6.81 (d, *J* = 8.0 Hz, 1H), 7.21 (d, *J* = 8.0 Hz, 1H), 7.15-7-20 (series of m, 4H); ^13^C NMR (CDCl_3_) δ 20.96, 34.92, 39.69, 115.62, 116.23, 116.39, 122.65, 125.43, 128.15, 128.17, 129.97, 131.79, 130.86, 134.35, 136.38, 136.96, 138.18, 146.02, 153.66, 169.90 Anal. Calcd for C_20_H_20_O_3_: C, 77.90; H, 6.54; Found: C, 78.05; H, 6.57.

**Compound 6**: ^1^H NMR (CDCl_3_) δ 2.34 (s, 3H) 3.35 (m, 4H), 5.06-5.14 (series of m, 4H), 5.94 (m, 2H), 6.89 (d, *J* = 8.4 Hz, 1H), 6.99-7-09 (series of m, 2H), 7.14 (d, *J* = 8.4 Hz, 1H), 7.31-7-39 (series of m, 2H); ^13^C NMR (CDCl_3_) δ 21.02, 34.90, 39.36, 115.86, 116.01, 116.63, 123.08, 127.43, 128.31, 129.24, 130.40, 131.25, 132.27, 132.76, 135.44, 135.58, 137.78, 148.50, 150.92, 169.65; Anal. Calcd for C_20_H_20_O_3_: C, 77.90; H, 6.54; Found: C, 78.00; H, 6.52.

**Compound 7.** To a solution of honokiol **2** (2 g, 7.5 mmol) in dry acetone (20 mL) potassium

carbonate (2.06 g, 15 mmol) was added under N_2_. The reaction mixture was stirred at rt for 10 min. Acetic anhydride (1.68 mL g, 16.5 mmol) was added and, after stirring at rt for 1 h, the solution was filtered and rotoevaporated to obtain a viscous oil that was purified by flash chromatography using dichloromethane: petroleum ether 1: 1 solution as eluent to obtain **compound 7** [S2]: (oil) (2.12 g, 81%) and 80 : 20 mixture of **compound 5** + **compound 6** (oil) (0.23 g, 10%).

**Compound 7**: ^1^H NMR (CDCl_3_) δ 2.05 (s, 3H), 2.27 (s, 3H), 3.30 (d, *J* = 6.8 Hz, 2H), 3.41 (d, *J* = 6.8 Hz, 2H), 5.07-5.15 (series of m, 4H), 5.96 (m, 2H), 7.02 (d, *J* = 8 Hz, 1H), 7.08 (d, *J* = 8.0 Hz, 1H), 7.17 (dd, *J* = 2.4, 8.0 Hz, 1H), 7.20 (d, *J* = 2.4 Hz, 1H), 7.26 (dd, *J* = 2.4, 8.0 Hz, 1H), 7.28 (d, *J* = 2.4, Hz, 1H); ^13^C NMR (CDCl_3_) δ 20.85, 20.90, 34.68, 39.63, 115.98, 115.40, 122.30, 122.77, 127.89, 128.68, 130.85, 130.90, 131.65, 133.74, 135.54, 135.69, 137.0, 138.24, 146.01, 148.35, 169.37, 169,62; Anal. Calcd for C_22_H_22_O_4_: C, 75.41; H, 6.33; Found: C, 75.44; H, 6.30.

**Magnolol-2-*O*-(2ʹ,3ʹ,4ʹ,6ʹ-tetra acetyl)-β-D-glucopyranoside (Magnolol mono glucopyranoside acetate)** A mixture of 2,ʹ,3ʹ,4ʹ,6ʹ-tetra-*O*-acetyl-α-D-glucopyranosyl bromide (5.06 g, 12.3 mmol, 1.4 equiv) in dry dichloromethane (50 mL) was added dropwise at rt to a solution of magnolol **1** (2.09 g, 7.8 mmol, 1 equiv) in 20 mL of a 1:1 solution of NaHCO_3_ (1.5 M) and KCl (1.5 M) in the presence of 18-crown-6 (3.51 g, 13 mmol, 1 equiv). After 5 hrs of reflux, the reaction was quenched with H_2_O and the organic phase was extracted twice with dichloromethane. The collected organic extracts were washed with HCl 0.1 N and dried over Na_2_SO_4_. Magnolol mono glucopyranoside acetate was purified by flash-chromatography (petroleum ether : ethyl ether 3:1) from the crude of reaction as a colourless oil (3.0 g, 64% yield). ^1^H NMR (CDCl_3_): δ 1.94 (3H, s), 2.00 (3H, s), 2.07 (3H, s), 2.14 (3H, s), 3.33 (2H, d, *J* = 6.4 Hz), 3.81-3.77 (1H, *m*), 4.14 (1H, dd, *J* = 2.4, 12.0 Hz), 4.24 (1H, dd, *J* = 5.2, 12.0 Hz), 5.15-5.01 (8H, series of m), 5.97-5.90 (2H, series of m), 6.86 (1H, d, *J* = 8.4 Hz), 6.96 (1H, d, *J* = 2 Hz), 7.06 (1H, dd, *J* = 2.4, 8 Hz), 7.08-7.11 (1H, *m*), 7.14 (2H, d, *J* = 1.2 Hz). ^13^C NMR (CDCl_3_): δ 20.1, 20.5, 20.6, 20.7, 30.9, 39.4, 61.8, 68.1, 70.6, 71.9, 72.6, 99.6, 115.6, 116.1, 117.1, 124.5, 128.2, 129.3, 129.4, 130.9, 132.1, 132.4, 136.2, 137.0, 137.7, 151.5, 151.8, 168.9, 169.4, 170.2, 170.6. Anal. Calcd for C_32_H_36_O_11_ C, 64.42; H, 6.08; Found C, 64.45; H, 6.11.

**Magnolol-2ʹ-*O*-β-D-glucopyranoside** (Magnolol mono glucopyranoside **8**) Magnolol mono glucopyranoside acetate (2.41g, 4.4 mmol) was solubilized in methanol (10 mL) and treated with sodium methoxide (22 mmol, 1.2g) for 5 hrs at rt. The reaction was quenched with a few drops of acetic acid at pH 6. After evaporation of the solvent, the solid was washed with ethyl ether and then with water to give 1.5 g of a white solid, characterized as magnolol mono glucopyranoside **8** (1.9 g, 80% yield), mp: 68-70 °C (lit.^[S3]^ 68.9 -70.1 °C); [α]^20^_546_ = -17.3 (c = 1, CHCl_3_). ^1^H NMR [(CD_3_)_2_CO]: δ 3.43-3.28 (6H, m), 3.55-3.50 (2H, m), 3.67 (1H, dd, *J* = 5.6, 12 Hz), 3.86 (1H, dd, *J* = 2.8, 12 Hz), 5.12-4.99 (5H, series of m), 6.12-5.95 (2H, m), 6.92 (1H, d, *J* = 8 Hz), 7.02 (1H, d, *J* = 2 Hz), 7.04 (1H, dd, *J* = 2, 8 Hz), 7.07 (1H, d, *J* = 2 Hz), 7.13 (1H, dd, *J* = 2.4, 8.4 Hz), 7.17 (1H, d, *J* = 8.4 Hz). ^13^C NMR [(CD_3_)_2_CO]: δ 39.0, 39.1, 61.7, 70.2, 73.6, 76.7, 76.9, 101.0, 114.5, 114.7, 114.9, 117.2, 128.3, 128.6, 128.7, 131.4, 131.5, 131.9, 133.6, 137.9, 138.2, 152.1, 153.2. ^1^H NMR [DMSO-*d6*]: δ 3.04-3.12 (2H, series of m), 3.25-3.45 (7H, series of m), 3.68 (1H, d, *J* = 11.6 Hz), 4.92 (1H, d, *J* = 7.6 Hz), 5.47-5.1 (4H, series of m), 5.92 (2H, m), 6.81 (1H, d, *J* = 8.0 Hz), 6.93 (1H, dd, *J* = 2.0 and 8.0 Hz), 7.01 (1H, d, *J* = 2.0 Hz), 7.03 (1H, d, *J* = 2.0 Hz), 7.08 (1H, dd, *J* = 2.0 and 8.4 Hz), 7.10 (1H, d, *J* = 8.4 Hz). ^13^C NMR [DMSO-*d6*]: δ 61.22, 70.13, 73.80, 76.89, 77.52, 79.62, 100.82, 114.81, 115.68, 115.99, 116.52, 125.95, 128.24, 128.52, 130.59, 131.93, 132.24, 132.79, 138.43, 138.78, 152.62, 153.16. Anal. Calcd for C_24_H_28_O_7_ C, 67.28; H, 6.59; Found C, 67.32; H, 6.61.

**Supplementary References**

S1. Yang, L., Wang, Z., Lei, H., Chen, R., Wang, X., Peng, Y. Neuroprotective glucosides of magnolol and honokiol from microbial-specific glycosylation. Tetrahedron 2014;70, 8244-51.

S2. Maioli M, Basoli V, Carta P, Fabbri D, Dettori MA, Cruciani S, Serra PA, Delogu G. Synthesis of magnolol and honokiol derivatives and their effect against hepatocarcinoma cells. PLoS ONE 2018; 13: e0192178.

S3. Yang TH, Ma YB, Geng CA, Yan DX, Huang XY, Li TZ, Zhang X, Chen JJ. Synthesis and biological evaluation of magnolol derivatives as melatonergic receptor agonists with potential use in depression. Eur J Med Chem. 2018;156: 381-393.
